# Aberrant allometric scaling of cortical folding in preterm-born adults

**DOI:** 10.1093/braincomms/fcac341

**Published:** 2022-12-26

**Authors:** Benita Schmitz-Koep, Aurore Menegaux, Juliana Zimmermann, Melissa Thalhammer, Antonia Neubauer, Jil Wendt, David Schinz, Christian Wachinger, Marcel Daamen, Henning Boecker, Claus Zimmer, Josef Priller, Dieter Wolke, Peter Bartmann, Christian Sorg, Dennis M Hedderich

**Affiliations:** Department of Diagnostic and Interventional Neuroradiology, School of Medicine, Technical University of Munich, Ismaninger Street 22, 81675 Munich, Germany; TUM-NIC Neuroimaging Center, School of Medicine, Technical University of Munich, Ismaninger Street 22, 81675 Munich, Germany; Department of Diagnostic and Interventional Neuroradiology, School of Medicine, Technical University of Munich, Ismaninger Street 22, 81675 Munich, Germany; TUM-NIC Neuroimaging Center, School of Medicine, Technical University of Munich, Ismaninger Street 22, 81675 Munich, Germany; Department of Diagnostic and Interventional Neuroradiology, School of Medicine, Technical University of Munich, Ismaninger Street 22, 81675 Munich, Germany; TUM-NIC Neuroimaging Center, School of Medicine, Technical University of Munich, Ismaninger Street 22, 81675 Munich, Germany; Department of Diagnostic and Interventional Neuroradiology, School of Medicine, Technical University of Munich, Ismaninger Street 22, 81675 Munich, Germany; TUM-NIC Neuroimaging Center, School of Medicine, Technical University of Munich, Ismaninger Street 22, 81675 Munich, Germany; Department of Diagnostic and Interventional Neuroradiology, School of Medicine, Technical University of Munich, Ismaninger Street 22, 81675 Munich, Germany; TUM-NIC Neuroimaging Center, School of Medicine, Technical University of Munich, Ismaninger Street 22, 81675 Munich, Germany; Department of Diagnostic and Interventional Neuroradiology, School of Medicine, Technical University of Munich, Ismaninger Street 22, 81675 Munich, Germany; TUM-NIC Neuroimaging Center, School of Medicine, Technical University of Munich, Ismaninger Street 22, 81675 Munich, Germany; Department of Diagnostic and Interventional Neuroradiology, School of Medicine, Technical University of Munich, Ismaninger Street 22, 81675 Munich, Germany; TUM-NIC Neuroimaging Center, School of Medicine, Technical University of Munich, Ismaninger Street 22, 81675 Munich, Germany; Lab for Artificial Intelligence in Medical Imaging, School of Medicine, Technical University of Munich, Ismaninger Street 22, 81675 Munich, Germany; Functional Neuroimaging Group, Department of Diagnostic and Interventional Radiology, University Hospital Bonn, Venusberg-Campus 1, 53127 Bonn, Germany; Department of Neonatology, University Hospital Bonn, Venusberg-Campus 1, 53127 Bonn, Germany; Functional Neuroimaging Group, Department of Diagnostic and Interventional Radiology, University Hospital Bonn, Venusberg-Campus 1, 53127 Bonn, Germany; Department of Diagnostic and Interventional Neuroradiology, School of Medicine, Technical University of Munich, Ismaninger Street 22, 81675 Munich, Germany; TUM-NIC Neuroimaging Center, School of Medicine, Technical University of Munich, Ismaninger Street 22, 81675 Munich, Germany; Department of Psychiatry, School of Medicine, Technical University of Munich, Ismaninger Street 22, 81675 Munich, Germany; Department of Psychology, University of Warwick, University Road, Coventry CV4 7AL, UK; Warwick Medical School, University of Warwick, University Road, Coventry CV4 7AL, UK; Department of Neonatology, University Hospital Bonn, Venusberg-Campus 1, 53127 Bonn, Germany; Department of Diagnostic and Interventional Neuroradiology, School of Medicine, Technical University of Munich, Ismaninger Street 22, 81675 Munich, Germany; TUM-NIC Neuroimaging Center, School of Medicine, Technical University of Munich, Ismaninger Street 22, 81675 Munich, Germany; Department of Psychiatry, School of Medicine, Technical University of Munich, Ismaninger Street 22, 81675 Munich, Germany; Department of Diagnostic and Interventional Neuroradiology, School of Medicine, Technical University of Munich, Ismaninger Street 22, 81675 Munich, Germany; TUM-NIC Neuroimaging Center, School of Medicine, Technical University of Munich, Ismaninger Street 22, 81675 Munich, Germany

**Keywords:** human brain development, cortical folding, universal scaling law, structural magnetic resonance imaging, preterm birth

## Abstract

A universal allometric scaling law has been proposed to describe cortical folding of the mammalian brain as a function of the product of cortical surface area and the square root of cortical thickness across different mammalian species, including humans. Since these cortical properties are vulnerable to developmental disturbances caused by preterm birth in humans and since these alterations are related to cognitive impairments, we tested (i) whether cortical folding in preterm-born adults follows this cortical scaling law and (ii) the functional relevance of potential scaling aberrances. We analysed the cortical scaling relationship in a large and prospectively collected cohort of 91 very premature-born adults (<32 weeks of gestation and/or birthweight <1500 g, very preterm and/or very low birth weight) and 105 full-term controls at 26 years of age based on the total surface area, exposed surface area and average cortical thickness measured with structural magnetic resonance imaging and surface-based morphometry. We found that the slope of the log-transformed cortical scaling relationship was significantly altered in adults (very preterm and/or very low birth weight: 1.24, full-term: 1.14, *P* = 0.018). More specifically, the slope was significantly altered in male adults (very preterm and/or very low birth weight: 1.24, full-term: 1.00, *P* = 0.031), while there was no significant difference in the slope of female adults (very preterm and/or very low birth weight: 1.27, full-term: 1.12, *P* = 0.225). Furthermore, offset was significantly lower compared with full-term controls in both male (very preterm and/or very low birth weight: −0.546, full-term: −0.538, *P* = 0.001) and female adults (very preterm and/or very low birth weight: −0.545, full-term: −0.538, *P* = 0.023), indicating a systematic shift of the regression line after preterm birth. Gestational age had a significant effect on the slope in very preterm and/or very low birth weight adults and more specifically in male very preterm and/or very low birth weight adults, indicating that the difference in slope is specifically related to preterm birth. The shape or tension term of the scaling law had no significant effect on cognitive performance, while the size of the cortex did. Results demonstrate altered scaling of cortical surface and cortical thickness in very premature-born adults. Data suggest altered mechanical forces acting on the cortex after preterm birth.

## Teaser

Using structural magnetic resonance imaging, Schmitz-Koep *et al*. observed aberrant allometric scaling of cortical surface and cortical thickness in preterm-born adults. Results suggest altered cortical folding in human prematurity.

## Graphical Abstract

**Graphical abstract:**
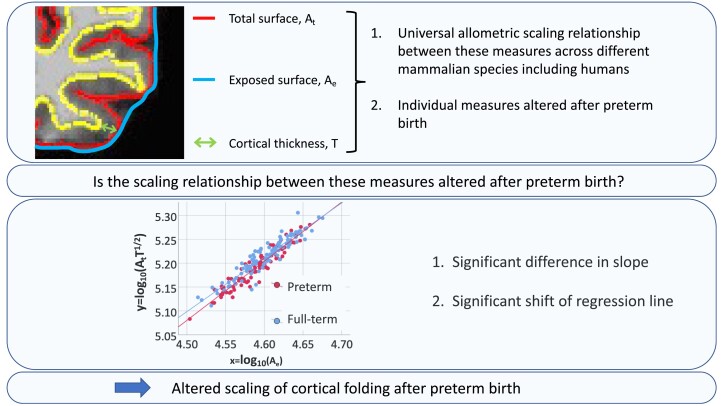

## Introduction

Cortical folding is a highly complex developmental and evolutional property of the mammalian brain, which allows for volume growth and surface area expansion despite restricted space in the skull.^1,2^ It is a milestone of mammalian—and particularly human—brain development influencing cognitive functions.^1,2^ However, folding mechanisms are not yet fully understood: while some hypotheses emphasize regional differences in tangential cortical expansion as a primary mechanism,^2,3^ others propose a tension-based theory highlighting the effect of tension along axons in white matter.^4,5^

Recently, a universal model of cortical folding has been proposed, showing that lissencephalic and gyrencephalic mammalian brains follow an allometric scaling law across different mammalian species, including humans.^6–8^ The concept of allometric scaling refers to a nonlinear relationship between biological measures.^9^ The model by Mota and Herculano-Houzel^6^ can be considered a basic principle of mammalian brain architecture and describes cortical folding as a power law relation between cortical thickness (T), exposed surface area (A_e_) and total surface area (A_t_) based on the physics of minimization of effective free energy: ${\text{At}}_{t}{\text{T}}^{\frac{1}{2}}={\text{kAe}}_{e}^{\alpha }$. The model predicts the scaling exponent α = 1.25. The only free parameter is *k*, or offset, which is adimensional.^6^ More, specifically, this means that a relative change in the product A_t_T^1/2^ (for example a relative change in T) links with a proportional relative change in A_e_^1.25^; one should note that such a relationship has several implications, for example, cortical folding scales rather with thickness and surface than with the number of neurones.^6^

Preterm birth (i.e. < 37 weeks of gestation) is associated with structural brain aberrations that last into adulthood, for example, alterations in cortical architecture, white matter integrity and subcortical structures.^10–22^ More specifically, several distinct cortical properties are altered in preterm-born adults compared with full-term (FT) controls: first, regionally reduced cortical surface area has been reported in preterm-born young adults.^19^ Second, we recently found regionally reduced T in a cohort of young adults born preterm.^16^ Third, we observed aberrant gyrification in this cohort.^11,15^ Furthermore, alterations in these cortical properties after preterm birth were associated with cognitive impairments.^11,15,16,19^ However, it remains unknown whether cortical folding after preterm birth follows the scaling law proposed by Mota and Herculano-Houzel^6^ and whether potential differences in scaling are functionally relevant in terms of cognitive performance.

Moreover, distinctive structural brain alterations after preterm birth indicate differential vulnerability of male and female individuals: for example, Benavides *et al*.^23^ found a sex-specific effect of gestational age (GA) on cortical volume in male infants born preterm at 12 months of age. Furthermore, at 8 years of age, males were particularly vulnerable to the adverse effects of preterm birth on white matter development.^24^ Since these measures influence cortical folding after preterm birth and because Kapellou *et al*.^25^ found a disrupted scaling relation between cortical surface area and cerebral volume in extremely preterm infants, which was particularly associated with the male sex, we also explored the interaction effect of sex and prematurity on the scaling law.

In the present study, we investigated whether the allometric scaling law of cortical folding holds in the case of preterm birth using structural MRI measures of 91 very premature-born adults [i.e. < 32 weeks of gestation and/or birth weight (BW) <1500 g] and 105 FT controls at 26 years of age. Furthermore, as alterations of individual properties of cortical folding, such as T and surface area, have been shown to be relevant for cognitive deficits after preterm birth,^16,19^ we also tested whether potential changes in the scaling relationship among these properties are also important for cognitive functioning in preterm-born adults.

## Methods

### Participants

Our study sample was previously described in:^16,26–28^ all participants were part of the Bavarian Longitudinal Study (BLS), a geographically defined, whole-population sample of neonatal at-risk children and healthy FT controls who were followed from birth into adulthood.^29,30^ Between January 1985 and March 1986, 682 infants were born very preterm (VP, < 32 weeks of gestation) and/or with very low birth weight (VLBW, BW <1500 g). From the initial 916 FT-born infants born in the same time period at the same obstetric hospitals who were alive at 6 years, 350 were randomly selected as control participants within the stratification variables of sex and family socioeconomic status in order to be comparable with the VP/VLBW sample. Informed consent from a parent and/or legal guardian was obtained. Of these infants, 411 VP/VLBW individuals and 308 controls were eligible for the 26-year follow-up assessment. 260 individuals from the VP/VLBW group and 229 controls participated in psychological assessments.^31^ All participants underwent screening for MRI-related exclusion criteria, which included (self-reported): claustrophobia, inability to lie still for > 30 min, unstable medical conditions (e.g. severe asthma), epilepsy, tinnitus, pregnancy, non-removable MRI-incompatible metal implants and a history of severe central nervous system trauma or disease that would impair further analysis of the data. The most frequent reason not to perform the MRI examination, however, was that participants declined to participate. Finally, 101 VP/VLBW individuals and 111 FT controls underwent MRI at 26 years of age (see Supplementary Fig. 1). MRI data acquisition took place at two sites: the Department of Neuroradiology, Klinikum rechts der Isar, Technische Universität München (*n* = 145) and the Department of Radiology, University Hospital of Bonn (*n* = 67). The study was carried out in accordance with the Declaration of Helsinki and was approved by the local ethics committees of the Klinikum rechts der Isar, Technische Universität München and the University Hospital Bonn. All study participants gave written informed consent and received travel expenses as well as a small payment for participation.

### Birth variables

As previously described,^12,27,32^ GA in weeks was determined from maternal reports on the first day of the last menstrual period and serial ultrasounds during pregnancy. When the difference between the methods was >2 weeks, clinical assessment at birth using the Dubowitz scoring system was applied.^33^ BW in grams was obtained from obstetric records. The duration of mechanical ventilation in days was computed from daily records by research nurses.

### Cognitive performance in adulthood

As previously described,^16,29,31^ global cognitive performance was assessed at the age of 26, prior to and independent of the MRI examination. Study participants were assessed using a short version of the ‘Wechsler Intelligenztest für Erwachsene’, the German adaptation of the Wechsler Adult Intelligence Scale, Third edition,^34^ which was carried out by trained psychologists who were blinded to group membership. This version included six subtests (vocabulary, similarities, letter-number-sequence, block design, matrix reasoning and digit symbol coding), which were used to derive full-scale intelligence quotient (IQ) estimates.^29,31^ Participants with missing data were excluded from the analysis of the functional relevance of scaling behaviour. Results were available for 88 VP/VLBW individuals and 103 FT individuals.

### MRI data acquisition

MRI data acquisition has been described previously:^12,22^ at both sites, Bonn and Munich, MRI data acquisition was performed on Philips Achieva 3T TX systems or Philips Ingenia 3T systems using an eight-channel SENSE head coil. Subject distribution among scanners: Bonn Achieva 3T: 5 VP/VLBW, 12 FT, Bonn Ingenia 3T: 33 VP/VLBW, 17 FT, Munich Achieva 3T: 60 VP/VLBW, 65 FT, Munich Ingenia 3T: 3 VP/VLBW, 17 FT. Across all scanners, sequence parameters were kept identical. Scanners were checked regularly to provide optimal scanning conditions and MRI physicists at the University Hospital Bonn and Klinikum Rechts der Isar regularly scanned imaging phantoms, to ensure within-scanner signal stability over time. The signal-to-noise ratio was not significantly different between scanners [one-way ANOVA with factor ‘Scanner-ID’ (Bonn 1, Bonn 2, Munich 1, Munich 2); F (3,182) = 1.84, *P* = 0.11]. A high-resolution T_1_-weighted 3D-magnetization prepared rapid acquisition gradient echo sequence (inversion time = 1300 ms, repetition time = 7.7 ms, echo time = 3.9 ms, flip angle = 15°, field of view = 256 mm × 256 mm, reconstruction matrix = 256 × 256 and reconstructed isotropic voxel size = 1 mm^3^) was acquired. All images were visually inspected for artefacts.

### MRI processing and surface-based morphometry to calculate measures of the scaling law

Images saved as DICOMs were converted to Nifti-format using dcm2nii.^35^ MRI data were processed using Freesurfer v6.0 (http://surfer.nmr.mgh.harvard.edu/). Total surface area A_t_ (pial surface area corrected for areas towards the corpus callosum), exposed surface area A_e_ (hull surface that envelops the pial surface but excludes sulcal regions corrected for areas towards the corpus callosum) and average T (distance between the white and the pial surface corrected for areas towards the corpus callosum) were extracted for each participant as described previously and global gyrification index was calculated as A_t_/A_e_.^8^ The Freesurfer ‘recon-all’ pipeline failed in three cases. Output quality was assessed using Freesurfer’s QA tools (https://surfer.nmr.mgh.harvard.edu/fswiki/QATools), and 13 subjects were excluded. Finally, 91 VP/VLBW subjects and 105 FT subjects were included in the analyses (see Supplementary Fig. 1).

### Statistical analysis

All statistical analyses were performed using IBM SPSS version 26 (IBM Corp., Armonk, NY, USA). Statistical significance was defined as *P* < 0.05. Age was not included as a covariate in our analysis, as VP/VLBW subjects and FT controls had the same mean age of 26 years (*P* = 0.274). To control for possible scanner effects, we applied ComBat, a technique that removes unwanted sources of scanner variability.^36^ ComBat-harmonized values of A_t_, A_e_ and T were used for all further analyses.

### Universal scaling law in preterm-born adults and comparison with full-term adults

To investigate the universal scaling law, we transformed to logarithmic coordinates, i.e. , x = log_10_(A_e_) and y = log_10_(A_t_T^1/2^) with α as a slope. First, to compare the slopes of VP/VLBW individuals and FT controls, we used multiple linear regression with sex and group (VP/VLBW versus FT) as categorical variables. 95% confidence intervals (CIs) were calculated. As a control analysis, we regressed out sex from *x* and *y* separately and repeated the multiple linear regression analysis with the residuals of *x* and *y* and with the group as a categorical variable.

Second, we compared the mean offset. Following Wang *et al*.^8^ we assumed a linear relationship with the slope of 1.25 as predicted by the theory with log(*k*) as the offset (K = log*At* − 1.25log*Ae* + 0.25log*T*^2^). We used multiple linear regression with sex and group as categorical variables.

Because there was a significant interaction effect of sex:group on the slope (*P* = 0.048), we repeated comparisons of slope and offset between VP/VLBW individuals and FT controls for male and female participants separately. We used multiple linear regression with the group as a categorical variable.

As a control analysis, we investigated the slope of the scaling law in a publicly available dataset of healthy young adults. We analysed data from 100 unrelated subjects aged 22–36 (46 males, 54 females) from the Human Connectome Project (HCP; www.humanconnectome.org; WU-Minn HCP Data—1200 Subjects; see Supplementary Analysis 1 and Fig. 2).

### Effect of birth variables on scaling behaviour

To assess the effect of birth variables on scaling behaviour, we first investigated the effect of GA, BW and ventilation on the slope within the VP/VLBW group. We used multiple linear regression with GA, BW and duration of ventilation as continuous variables and sex as a covariate of no interest. Results were corrected for multiple comparisons using the Benjamini–Hochberg procedure.^37^

Because there was a sex-specific effect of preterm birth on the slope, we repeated this analysis for male and female VP/VLBW subjects separately. Results were corrected for multiple comparisons using the Benjamini–Hochberg procedure.^37^

Second, we investigated the effect of GA, BW and duration of ventilation on the offset (calculated using the theoretically predicted slope of 1.25) using multiple linear regression with GA, BW and duration of ventilation as continuous variables and sex as a covariate of no interest. Results were corrected for multiple comparisons using the Benjamini–Hochberg procedure.^37^

### Functional relevance of scaling behaviour

To assess the functional relevance of scaling behaviour, we used the independent morphological measures offset, or tension term (K = log*A*_*t*_ − 1.25log*A*_*e*_ + 0.25log*T*^2^), the isometric term (I = log*A*_*t*_ + log*A*_*e*_ + log*T*^2^) and shape term (S = $\frac{3}{2}$log*A*_*t*_ + $\frac{3}{4}$log*A*_*e*_ − $\frac{9}{4}$log*T*^2^) proposed by Wang *et al*.^38^ We used multiple linear regression with sex and group as categorical variables to compare offset, isometric term and shape term between VP/VLBW individuals and FT controls.

To analyse functional relevance, we used multiple linear regression with offset, isometric term and shape term as a independent variables, full-scale IQ as dependent variable and sex as a covariate of no interest in the VP/VLBW group.

## Results

### Sample characteristics

Table 1 presents group demographic and clinical background variables. There was no significant difference between the VP/VLBW group and the FT group regarding sex (*P* = 0.657) or age at scanning (*P* = 0.274). By design of the study, VP/VLBW subjects had significantly lower GA (*P* < 0.001) and lower BW (*P* < 0.001). Furthermore, VP/VLBW individuals had significantly lower full-scale IQ (*P* < 0.001), verbal IQ (*P* = 0.001), performance IQ (*P* < 0.001), total surface area (*P* = 0.001), exposed surface area (*P* = 0.023) and the global gyrification index (*P* < 0.001). Global T was not significantly lower in the VP/VLBW group compared with the FT group (*P* = 0.165). Results were very similar after correcting for scanner effects using ComBat harmonization. VP/VLBW individuals had a significantly lower total surface area (*P* = 0.001), exposed surface area (*P* = 0.021) and global gyrification index (*P* < 0.001), while global T was not significantly lower in the VP/VLBW group compared with the FT group (*P* = 0.152). Tables 2 and 3 present group demographic and clinical background variables for male and female sex separately. There was no significant difference between the male VP/VLBW group and the FT group, as well as between the female VP/VLBW group and the FT group, regarding sex (male: *P* = 0.805, female *P* = 167). Compared with male and female FT subjects, male and female VP/VLBW subjects had significantly lower GA (male: *P* < 0.001, female: *P* < 0.001), lower BW (male: *P* < 0.001, female: *P* < 0.001), lower full-scale IQ (male: *P* = 0.003, female: *P* = 0.001), verbal IQ (male: *P* = 0.001, female *P* = 0.010), performance IQ (male: *P* = 0.001, female *P* = 0.005), total surface area (male: *P* = 0.001, female *P* = 0.025) and global gyrification index (male: *P* = 0.001, female *P* = 0.002), respectively. The exposed surface area was significantly lower in male VP/VLBW subjects compared with male FT subjects (*P* = 0.024). There was no significant difference in exposed surface area in female subjects (*P* = 0.168). Global T was not significantly different in both male and female VP/VLBW subjects compared with male and female FT subjects, respectively (male: *P* = 0.150, female: *P* = 0.636). Results were very similar after correcting for scanner effects using ComBat harmonization. Male and female VP/VLBW subjects had a significantly lower total surface area (male: *P* = 0.001, female *P* = 0.015) and global gyrification index (male: *P* = 0.001, female *P* = 0.002) compared with male and female FT controls. Exposed surface area was significantly lower in male VP/VLBW subjects compared with male FT subjects (*P* = 0.029). There was no significant difference in exposed surface area in female subjects (*P* = 0.126). Global T was not significantly different in both male and female VP/VLBW subjects compared with male and female FT subjects, respectively (male: *P* = 0.156, female: *P* = 0.580).

**Table 1:** Demographical, clinical and cognitive data of all subjects

	VP/VLBW (*n* = 91)			FT (*n* = 105)			*P*-value
	Mean/*n*	SD	Range	Mean/*n*	SD	Range	
Sex (male/female)	50/41	–	–	61/44	–	–	0.657
Age (years)	26.7	± 0.6	25.7–28.3	26.8	± 0.8	25.5–28.9	0.274
GA (weeks)	30.6	± 2.2	25–36	39.7	± 1.1	37–42	**<0**.**001**
BW (g)	1328	± 309	630–2000	3396	± 449	2120–4670	**<0**.**001**
Ventilation (days)	11.5	± 17.3	0–81	–	–	–	–
Full-scale IQ (a.u.)^a^	94.1	± 12.9	64–131	102.2	± 11.8	77–130	**<0**.**001**
Verbal IQ (a.u.)^a^	98.5	± 13.8	62–137	105.5	± 14.2	77–143	**0**.**001**
Performance IQ (a.u.)^a^	90.0	± 13.9	56–118	98.2	± 10.3	69–125	**<0**.**001**
A_t_ (mm^2^)	97860.2	± 9125.1	76498.4–120288.7	102402.9	± 9155.4	83071.6–125209.4	**0**.**001**
A_e_ (mm^2^)	38985.6	± 2856.3	31807.4–45819.4	39948.6	± 2986.9	32588.7–47640.7	**0**.**023**
T (mm)	2.54	± 0.09	2.34–2.82	2.56	± 0.10	2.31–2.85	0.165
Gyrification index	2.51	± 0.09	2.32–2.72	2.56	± 0.08	2.34–2.84	**<0**.**001**
ComBat-harmonized:							
A_t_ (mm^2^)	97892.4	± 8961.7	76771.0–119265.6	102434.5	± 9131.3	83406.6–124159.0	**0**.**001**
A_e_ (mm^2^)	38969.0	± 2827.1	31893.0–45580.8	39948.3	± 3010.5	32677.8–47348.4	**0**.**021**
T (mm)	2.54	± 0.09	2.34–2.82	2.56	± 0.10	2.33–2.84	0.152
Gyrification index	2.51	± 0.09	2.32–2.72	2.56	± 0.08	2.34–2.84	**<0**.**001**

Statistical comparisons: sex with χ^2^ statistics; age, GA, BW, full-scale IQ, verbal IQ, performance IQ, A_t_, A_e_, T, gyrification index and ComBat-harmonized values with two sample *t*-tests. Bold letters indicate statistical significance defined as *P* < 0.05.

^a^ Data are based on 88 VP/VLBW individuals and 103 FT individuals. Abbreviations: SD = standard deviation.

**Table 2:** Demographical, clinical and cognitive data of male subjects

	VP/VLBW (*n* = 50)			FT (*n* = 61)			*P*-value
	Mean/*n*	SD	Range	Mean/*n*	SD	Range	
Age (years)	26.7	± 0.5	25.8–28.0	26.8	± 0.8	25.6–28.9	0.805
GA (weeks)	30.5	± 2.0	27–36	39.7	± 1.0	37–42	**<0**.**001**
BW (g)	1389	± 314	750–2000	3440	± 482	2120–4670	**<0**.**001**
Ventilation (days)	10.3	± 16.1	0–79	–	–	–	–
Full-scale IQ (a.u.) ^a^	95.4	± 12.2	68–125	102.8	± 13.1	77–130	**0**.**003**
Verbal IQ (a.u.) ^a^	100.8	± 13.4	64–130	107.0	± 15.1	79–143	**0**.**001**
Performance IQ (a.u.) ^a^	89.8	± 12.2	61–114	97.7	± 11.5	69–125	**0**.**001**
A_t_ (mm^2^)	102819.4	± 7359.0	90107.3–120288.7	107502.4	± 6893.2	95358.0–125209.4	**0**.**001**
A_e_ (mm^2^)	40634.4	± 2093.9	37501.1–45819.4	41592.2	± 2280.6	36581.9–47640.7	**0**.**024**
T (mm)	2.54	± 0.09	2.35–2.72	2.57	± 0.09	2.37–2.85	0.150
Gyrification index	2.53	± 0.09	2.32–2.72	2.58	± 0.08	2.44–2.84	**0**.**001**
ComBat-harmonized							
A_t_ (mm^2^)	102988.8	± 7025.2	91324.8–119265.6	107505.1	± 6952.9	95376.9–124158.9	**0**.**001**
A_e_ (mm^2^)	40652.3	± 2014.2	37747.3–45580.8	41582.0	± 2334.9	36665.0–47348.4	**0**.**029**
T (mm)	2.54	± 0.09	2.34–2.72	2.57	± 0.09	2.36–2.84	0.156
Gyrification index	2.53	± 0.09	2.32–2.72	2.58	± 0.08	2.44–2.84	**0**.**001**

Statistical comparisons: sex with χ^2^ statistics; age, GA, BW, full-scale IQ, verbal IQ, performance IQ, A_t_, A_e_, T, gyrification index and ComBat-harmonized values with two sample *t*-tests. Bold letters indicate statistical significance defined as *P* < 0.05.

^a^ Data are based on 49 VP/VLBW individuals and 61 FT individuals. Abbreviations: SD = standard deviation.

**Table 3:** Demographical, clinical and cognitive data of female subjects

	VP/VLBW (*n* = 41)			FT (*n* = 44)			
	Mean/*n*	SD	Range	Mean/*n*	SD	Range	*P*-value
Age (years)	26.7	± 0.7	25.7–28.3	26.9	± 0.7	25.5–28.3	0.167
GA (weeks)	30.7	± 2.4	25–36	39.7	± 1.1	37–42	**<0**.**001**
BW (g)	1255	± 290	630–1960	3335	± 397	2450–4200	**<0**.**001**
Ventilation (days)	12.9	± 18.7	0–81	–	–	–	–
Full-scale IQ (a.u.) ^a^	92.5	± 13.8	64–131	101.2	± 9.6	80–124	**0**.**001**
Verbal IQ (a.u.) ^a^	95.6	± 13.9	62–137	103.5	± 12.6	77–133	**0**.**010**
Performance IQ (a.u.) ^a^	90.3	± 16.0	56–118	98.8	± 8.4	78–114	**0**.**005**
A_t_ (mm^2^)	91812.3	± 7264.8	76498.4–112532.8	95333.2	± 6956.7	83071.6–113278.4	**0**.**025**
A_e_ (mm^2^)	36974.7	± 2333.1	31807.4–42742.2	37669.9	± 2275.1	32588.7–42117.0	0.168
T (mm)	2.54	± 0.10	2.34–2.82	2.55	± 0.11	2.31–2.80	0.636
Gyrification index	2.48	± 0.07	2.34–2.63	2.53	± 0.07	2.34–2.69	**0**.**002**
ComBat-harmonized							
A_t_ (mm^2^)	91677.3	± 6941.4	76771.0–111348.5	95404.7	± 6868.0	83406.6–112235.7	**0**.**015**
A_e_ (mm^2^)	36916.1	± 2269.8	31893.0–42501.7	37683.3	± 2297.3	32677.8–41922.0	0.126
T (mm)	2.54	± 0.10	2.34–2.82	2.55	± 0.11	2.33–2.79	0.580
Gyrification index	2.48	± 0.07	2.34–2.63	2.53	± 0.07	2.34–2.69	**0**.**002**

Statistical comparisons: sex with χ^2^ statistics; age, GA, BW, full-scale IQ, verbal IQ, performance IQ, A_t_, A_e_, T, gyrification index and ComBat-harmonized values with two sample *t*-tests. Bold letters indicate statistical significance defined as *P* < 0.05.

^a^ Data are based on 39 VP/VLBW individuals and 42 FT individuals. Abbreviations: SD = standard deviation.

### Universal scaling law in preterm-born adults

**Figure 1 Comparison of scaling behaviour and of offset between preterm-born adults and FT controls.**
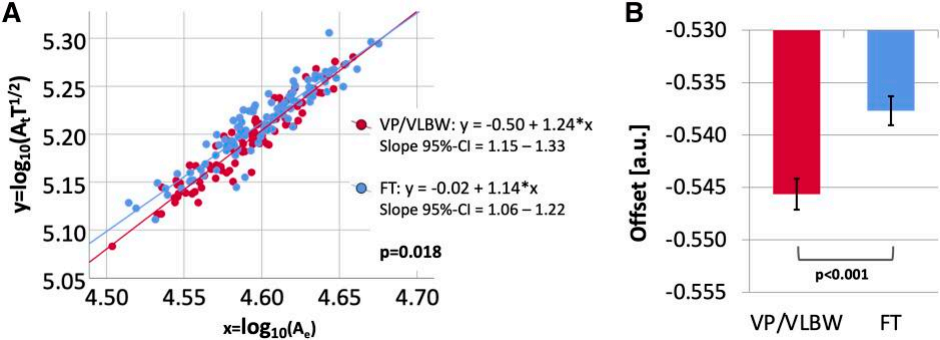
(**A**) The relationship between x = log_10_(A_e_) and y = log_10_(A_t_T^1/2^) is shown as a scatter plot. Linear regression lines and equations for the VP/VLBW (red) and FT (blue) group as well as 95% CI intervals for the slope and the *P*-value from the multiple linear regression analysis were added. There was a significant effect of group on the slope [VP/VLBW: 1.24 (95% CI = 1.15–1.33), FT: 1.14 (95% CI = 1.06–1.22); *F* (1189) = 5.655, *P* = 0.018]. Bold letters indicate statistical significance. (**B**) Using the theoretically predicted slope of 1.25, estimated marginal means for the VP/VLBW (red) and FT (blue) group are shown as bar charts with SE as error bars and the *P*-value from the multiple linear regression analysis was added. There was a significant effect of group on offset [VP/VLBW: −0.546, FT: −0.538; *F* (1192) = 15.659, *P* < 0.001]. Bold letters indicate statistical significance.

**Figure 2 Comparison of scaling behaviour and of offset between male preterm-born adults and male FT controls.**
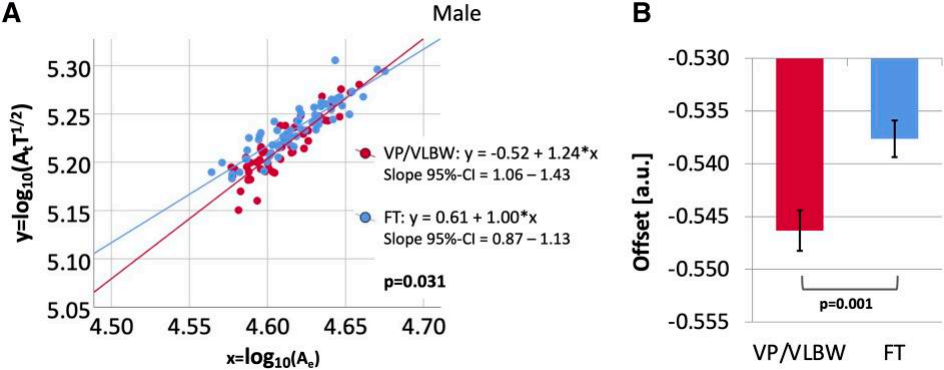
(**A**) The relationship between x = log_10_(A_e_) and y = log_10_(A_t_T^1/2^) is shown as a scatter plot. Linear regression lines and equations for the male VP/VLBW (red) and FT (blue) group as well as 95% CI intervals for the slope and the *P*-value from the multiple linear regression analysis were added. There was a significant effect of group on the slope in male participants [VP/VLBW: 1.24 (95% CI = 1.06–1.43), FT: 1.00 (95% CI = 0.87–1.13); *F* (1107) = 4.782, *P* = 0.031]. Bold letters indicate statistical significance. (**B**) Using the theoretically predicted slope of 1.25, estimated marginal means for the male VP/VLBW (red) and FT (blue) group are shown as bar charts with SE as error bars and the *P*-value from the multiple linear regression analysis was added. There was a significant effect of group on offset in male participants [VP/VLBW: −0.546, FT: −0.538; *F* (1109) = 11.336, *P* = 0.001]. Bold letters indicate statistical significance.

To compare the slopes of VP/VLBW individuals and FT controls, we used multiple linear regression and found a significant effect of group (VP/VLBW versus FT) on the slope [VP/VLBW: 1.24 (95% CI = 1.15–1.33), FT: 1.14 (95% CI = 1.06–1.22); *F* (1189) = 5.655, *P* = 0.018]. The theoretically predicted slope of 1.25 is not within the 95% CI of the slope for FT controls. However, the 95% CI of the slope for FT controls overlaps with the 95% CI of the slope for subjects in the age range 22–36 years from the HCP [slope = 1.22 (95% CI = 1.14–1.29); see Supplementary Analysis 1 and Fig. 2]. Furthermore, there was a significant interaction effect of sex:group on the slope [*F* (1189) = 3.956, *P* = 0.048]. Regressing out sex from *x* and *y* separately as a control analysis, there was still a significant effect of the group on the slope [*F* (1192) = 6.239, *P* = 0.013]. Figure 1A shows a scatter plot with regression lines comparing the logarithmic-transformed universal scaling law between individuals of the VP/VLBW (red) and the FT group (blue). Second, we used multiple linear regression to compare the mean offset of the scaling law between VP/VLBW individuals and FT controls. There was a significant main effect of the group on offset, considering the theoretically predicted slope of 1.25 [VP/VLBW: −0.546, FT: −0.538; *F* (1,192) = 15.659, *P* < 0.001]. Figure 1B shows the estimated marginal means of offset in VP/VLBW and FT subjects with standard error (SE) as error bars. There was no significant interaction effect of sex:group on offset [*F* (1,192) = 0.127, *P* = 0.722].

Because there was a significant interaction effect of sex:group on the slope, we repeated comparisons of slope and offset between VP/VLBW individuals and FT controls for male and female participants separately. In male participants, there was a significant effect of group on the slope [VP/VLBW: 1.24 (95% CI = 1.06–1.43), FT: 1.00 (95% CI = 0.87–1.13); *F* (1107) = 4.782, *P* = 0.031]. The theoretically predicted slope of 1.25 is not within the 95% CI of the slope for male FT controls. However, the 95% CI of the slope for male FT controls overlaps with the 95% CI of the slope for subjects in the age range 22–36 years from the HCP [slope = 1.21 (95% CI = 1.08–1.34); see Supplementary Analysis 1 and Fig. 2]. Figure 2A shows a scatter plot with regression lines comparing the logarithmic-transformed universal scaling law between male individuals of the VP/VLBW (red) and the FT group (blue). There was a significant main effect of the group on offset in male participants, considering the theoretically predicted slope of 1.25 [VP/VLBW: −0.546, FT: −0.538; *F* (1109) = 11.336, *P* = 0.001]. Figure 2B shows the estimated marginal means of offset in male VP/VLBW and FT subjects with SE as error bars.

**Figure 3 Comparison of scaling behaviour and of offset between female preterm-born adults and female FT controls.**
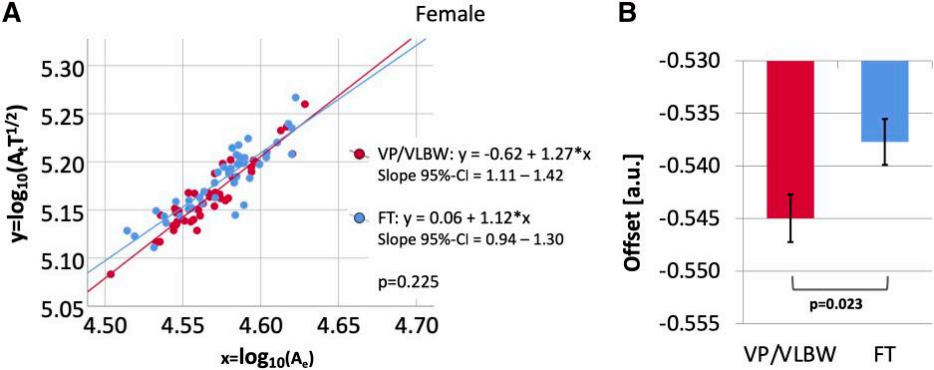
(**A**) The relationship between x = log_10_(A_e_) and y = log_10_(A_t_T^1/2^) is shown as a scatter plot. Linear regression lines and equations for the female VP/VLBW (red) and FT (blue) group as well as 95% CI intervals for the slope and the *P*-value from the multiple linear regression analysis were added. There was no significant effect of group on the slope in female participants [VP/VLBW: 1.27 (95% CI = 1.11–1.42), FT: 1.12 (95% CI = 0.94–1.30); *F* (1,81) = 1.492, *P* = 0.225]. Bold letters indicate statistical significance. (**B**) Using the theoretically predicted slope of 1.25, estimated marginal means for the female VP/VLBW (red) and FT (blue) group are shown as bar charts with SE as error bars and the *P*-value from the multiple linear regression analysis was added. There was a significant effect of group on offset in female participants [VP/VLBW: −0.545, FT: −0.538; *F* (1,83) = 5.336, *P* = 0.023]. Bold letters indicate statistical significance.

In female participants, there was no significant effect of group on the slope [VP/VLBW: 1.27 (95% CI = 1.11–1.42), FT: 1.12 (95% CI = 0.94–1.30); *F* (1,81) = 1.492, *P* = 0.225]. Figure 3A shows a scatter plot with regression lines comparing the logarithmic-transformed universal scaling law between female individuals of the VP/VLBW (red) and the FT group (blue). There was a significant main effect of group on offset in female participants, considering the theoretically predicted slope of 1.25 [VP/VLBW: −0.545, FT: −0.538; *F* (1,83) = 5.336, *P* = 0.023]. Figure 3B shows the estimated marginal means of offset in female VP/VLBW and FT subjects with SE as error bars.

In summary, preterm birth had a significant effect on the scaling of the cortical surface and T in adults. The exponent of the scaling law was significantly different in male preterm-born adults. Furthermore, offset was significantly lower in male and female preterm-born adults compared with FT controls, suggesting a systematic shift in the relationship between cortical surface and thickness.

### Effect of birth variables on scaling behaviour

We first assessed the effect of birth variables on the slope in the VP/VLBW group using multiple linear regression analysis. After false discovery rate correction using the Benjamini–Hochberg procedure,^37^ there was a significant effect of GA on the slope [*F* (1,83) = 19.748, *P* < 0.001]. There was no significant effect of BW or duration of ventilation on the slope [*F* (1,83) = 0.164, *P* = 0.686 and *F* (1,83) = 0.002, *P* = 0.963, respectively].

Because there was a sex-specific effect of preterm birth on the slope, we repeated this analysis for male and female VP/VLBW subjects separately. After false discovery rate correction using the Benjamini–Hochberg procedure,^37^ there was a significant effect of GA on the slope [*F* (1,43) = 10.947, *P* = 0.002] in the group of male VP/VLBW subjects. There was no significant effect of BW or duration of ventilation on the slope [*F* (1,43) = 0.518, *P* = 0.476 and *F* (1,43) = 0.163, *P* = 0.689, respectively). In the group of female VP/VLBW subjects, there was no significant effect of GA, BW or duration of ventilation on the slope after false discovery rate correction using the Benjamini–Hochberg procedure^37^ [*F* (1,34) = 5.353, *P* = 0.027, *F* (1,34) = 0.037, *P* = 0.848 and *F* (1,34) = 2.079, *P* = 0.159, respectively].

Second, we investigated the effect of birth variables on the offset using multiple linear regression analysis. This analysis was restricted to VP/VLBW subjects since offset was significantly lower in VP/VLBW subjects regardless of sex. After false discovery rate correction using the Benjamini–Hochberg procedure,^37^ there was no significant effect of GA, BW or duration of ventilation on offset [*F* (1,86) = 0.247, *P* = 0.620; *F* (1,86) = 4.312, *P* = 0.041; and *F* (1,86) = 0.198, *P* = 0.657, respectively].

In summary, GA had a significant effect on the exponent of the universal scaling law in male preterm-born adults, indicating that the difference is specifically related to preterm birth.

### Functional relevance of scaling behaviour

To assess the functional relevance of scaling behaviour, we analysed the effect of the independent morphological measures offset (tension term), isometric term and shape term on cognitive performance in the VP/VLBW group. Offset was significantly lower in VP/VLBW adults compared with FT controls. Furthermore, there was a significant difference in the isometric term between groups [VP/VLBW: 10.388, FT: 10.425; *F* (1,192)= 12.937, *P* < 0.001]. There was no significant difference in the shape term between VP/VLBW adults and FT controls [VP/VLBW: 9.105, FT: 9.128; *F* (1,192)= 2.345, *P* = 0.127]. There was no significant interaction effect of sex:group on offset, the isometric term [*F* (1,192) = 0.113, *P* = 0.738] or shape term [*F* (1,192) = 0.069, *P* = 0.794].

After false discovery rate correction using the Benjamini–Hochberg procedure,^37^ there was a significant effect of the isometric term on full-scale IQ [*F* (1,83) = 15.266, *P* < 0.001]. There was no significant effect of offset and the shape term on full-scale IQ [*F* (1,83) = 0.838, *P* = 0.363 and *F* (1,83) = 0.942, *P* = 0.335, respectively].

In summary, while the size of the cortex had a significant effect on cognitive performance in preterm-born adults, its shape or tension had no significant effect.

## Discussion

Based on structural MRI, we found a significant effect of preterm birth on the scaling of the cortical surface and T in adults. The exponent of the scaling law was significantly different in male preterm-born adults, which was associated with GA. Both male and female adults born preterm showed significantly lower offsets compared with FT controls, suggesting a systematic shift in the relationship between cortical surface and thickness. The shape or tension term of the scaling law had no significant effect on cognitive performance, while the size of the cortex did. Hence, altered neurodevelopment after preterm birth leads to differences in the allometric scaling of cortical folding.

### Universal scaling law of cortical folding in preterm-born adults

We found a significant effect of prematurity on the exponent of the universal scaling law, specifically in male participants, implying fundamental differences in the physical model proposed by Mota and Herculano-Houzel.^6^ GA had a significant effect on the slope in preterm-born adults and more specifically in male preterm-born adults, indicating that this difference in the allometric scaling principles is specifically related to preterm birth. Both male and female preterm-born adults showed significantly lower offsets compared with FT controls, indicating a systematic shift after preterm birth, which implies that other parameters, such as changes in the mechanical forces acting on the cortex, may differ.

First, previous results of allometric scaling of brain growth after preterm birth are heterogenous: in line with our results, Kapellou *et al*.^25^ found a disrupted scaling relation in extremely preterm infants indicated by a lower scaling exponent. Increasing prematurity and male sex were associated with a lower scaling exponent. In contrast, Paul *et al*.^39^ found no influence of GA on the power law scaling relationship in preterm infants. However, first, these studies investigated infants, while the present study analysed young adults; second, the scaling law tested in these two studies was based on the relationship between surface area and cerebral volume, while Mota and Herculano-Houzel^6^ described the degree of folding as a function of the product of surface area and the square root of T; and third, no control groups were used to analyse group differences. Using the same scaling law as Mota and Herculano-Houzel,^6^ Wang *et al*.^8^ investigated the effects of sex, age and the presence of Alzheimer’s disease on cortical folding. While healthy male and female brains scaled in the same way, patients with Alzheimer’s disease had a possible change in the exponent compared with controls.^8^ In the present study, we could not confirm the theoretically predicted exponent of 1.25 of the universal scaling law proposed by Mota and Herculano-Houzel^6^ in all FT controls and in male FT controls, since 1.25 was not within the 95% CI of the slope for all FT controls and for male FT controls. A potential reason might be scanner effects. However, both for all FT controls and for male FT controls, the 95% CI of the slope overlapped with the 95% CI of the slope for healthy young adults aged 22–36 years from the HCP (see Supplementary Analysis 1 and Fig. 2). Nevertheless, controlling for scanner effects, the present study allows to compare the scaling law across groups (VP/VLBW versus FT), which is the main aim of this study.

Second, we found a significantly lower offset in preterm-born adults, indicating a systematic shift of the regression line of the logarithmic-transformed universal scaling law. In healthy subjects aged 4–94 years, ageing introduced a systematic shift in the offset of the scaling law.^8^ Furthermore, patients with Alzheimer’s disease had a lower offset compared with controls.^8^ Therefore, a lower offset after preterm birth could be interpreted as accelerated maturation, which is in line with our previous results suggesting an increased risk for accelerated brain ageing in human prematurity.^40^ Underlying mechanisms might be changes in the mechanical forces acting on the cortex, as offset seems to be a function of the internal tensions and external pressure applied to the grey matter.^8^ Hence, the lower offset may be interpreted as a slackening of white matter axonal tension or a decrease in white matter axonal density.^7^ In line with this interpretation, we recently found reduced apparent fibre density in numerous white matter tracts of preterm-born adults.^18^ However, cross-sectional data cannot answer questions regarding brain development. Therefore, results have to be interpreted with care and longitudinal studies across different age groups are needed to investigate brain ageing after preterm birth.

Third, the exponent of the scaling law was significantly different in male preterm-born adults, which was associated with GA. Previous studies also indicate differential vulnerability of male and female individuals after preterm birth: for example, male sex was associated with an increased risk for poorer neurologic outcome, such as a higher risk of severe disability, impaired neurodevelopment with lower scores for cognitive functioning and higher mortality.^41,42^ Furthermore, sex differences were found in structural brain alterations after preterm birth, such as cortical volume and white matter development, as well as in functional connectivity, as very preterm boys had greater alterations in resting neurophysiological network communication than girls.^23,24,43^ In line with our results, Kapellou *et al*.^25^ found that the male sex was associated with a lower scaling exponent in extremely preterm infants.

In summary, scaling of the cortical surface and T are different in preterm-born adults compared with controls. The exponent of the scaling law was significantly different in male preterm-born adults, implying fundamental differences in the physical model. Furthermore, there was a systematic shift in the relationship between cortical surface and thickness in both male and female preterm-born adults, possibly due to changes in the mechanical forces acting on the cortex.

### Functional relevance of scaling behaviour

We found a significant effect of the isometric term, which carries information about the size of the cortex and is highly correlated with grey matter volume,^38^ on cognitive performance. This result is in line with previous studies linking decreased grey matter volume after preterm birth with cognitive impairments.^44^ However, offset (tension term), which can be interpreted as tension/pressure applied to the cortical tissue^7,8,38^ and shape term had no significant effect on cognitive performance. In general, alterations of cortical macro-architecture (beyond volume) after preterm birth are associated with impaired cognitive performance: for example, reduced surface area was associated with reduced IQ scores in VLBW young adults.^19^ Furthermore, reduced T in the left hemisphere was associated with lower full-scale IQ in the cohort investigated in the present study.^16^ Lastly, gyrification and cortical complexity were linked with reduced full-scale IQ in the same cohort.^11,15^ A possible reason might be that we investigated a scaling law of global cortical folding in the present study, while associations might be regionally specific.

### Strengths and limitations

One of the strengths of our study is that a relevant impact of age is excluded, as VP/VLBW subjects and FT controls were examined in a narrow age range, with the same mean age of 26 years at the time of the MRI scan.

Furthermore, the study has the strength of a large sample (91 VP/VLBW and 105 FT adults).

A limitation of the present study is the bias of the current sample towards VP/VLBW adults with less severe neonatal complications, less functional impairments and higher IQ since participants with more birth complications in the initial BLS sample were more likely to be excluded in initial screening for MRI due to the exclusion criteria for MRI. Thus, the reported differences are conservative estimates of true differences. However, our final sample was still representative of the full cohort in terms of GA and BW, as these values were not significantly different in VP/VLBW subjects with MRI data compared with subjects without MRI data (see Supplementary Table 1).

## Conclusions

In conclusion, altered neurodevelopment after preterm birth leads to differences in allometric scaling of cortical surface and T with sex-specific effects on the exponent in male preterm-born adults and a systematic shift in both sexes, indicating altered mechanical forces acting on the cortex after preterm birth. Results suggest altered cortical folding in human prematurity.

## Supplementary Material

fcac341_Supplementary_Data

## Data Availability

Patient data used in this study are not publicly available but stored by the principal investigators of the BLS.

## Abbreviations

A_e_ =: exposed surface area
A_t_ =: total surface area
BLS =: Bavarian longitudinal study
BW =: birth weight
CI =: confidence interval
FOV =: field of view
FT =: full-term
GA =: gestational age
MPRAGE =: magnetization prepared rapid acquisition gradient echo
MRI =: magnetic resonance imaging
SE =: standard error
T =: cortical thickness
TE =: echo time
TI =: inversion time
TR =: repetition time
VLBW =: very low birth weight
VP =: very preterm
VP/VLBW =: very preterm and/or very low birth weight
