# Supplementary material for: Aberrant allometric scaling of cortical folding in preterm-born adults
Source: Brain Commun. 2022 Dec 26;5(1):fcac341. doi: 10.1093/braincomms/fcac341 (PMC9830984; doi:10.1093/braincomms/fcac341)
Supplement: fcac341_Supplementary_Data [file fcac341_supplementary_data.docx]

**- Supplement -**

**Supplementary Figure 1: Participants of the Bavarian Longitudinal Study**

**
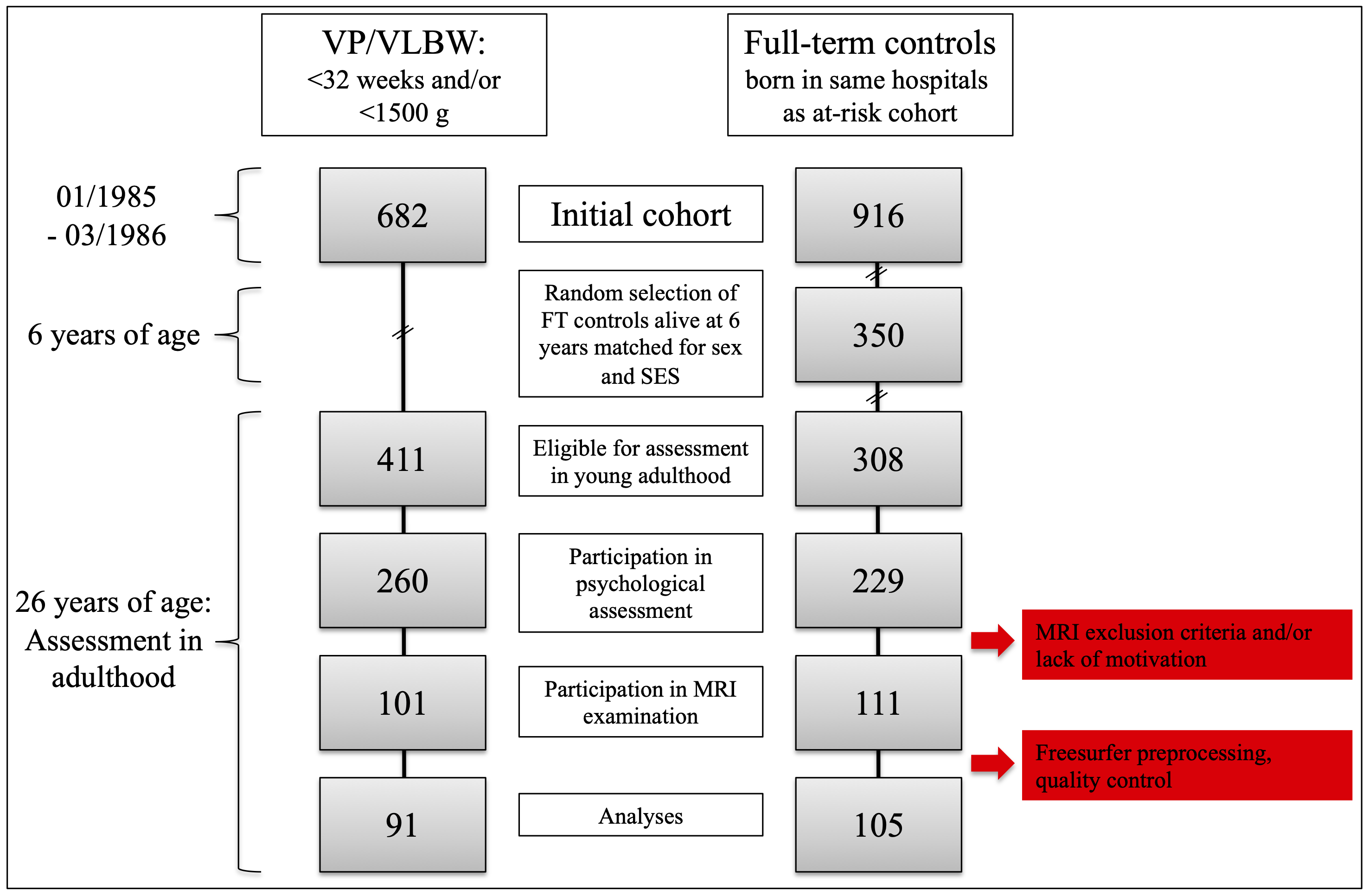
**

Flowchart of the participants of the Bavarian Longitudinal Study.

Abbreviations: MRI, magnetic resonance imaging; SES, socioeconomic status; VP/VLBW, very preterm and/or very low birth weight.

**Supplementary Analysis 1: Scaling law in a publicly available dataset of healthy young adults**

As a control analysis, we investigated the slope of the scaling law in a publicly available dataset of healthy young adults. We analyzed data from 100 unrelated subjects aged 22-36 years (46 males, 54 females) from the Human Connectome Project (HCP; www.humanconnectome.org; WU-Minn HCP Data - 1200 Subjects). We used preprocessed data provided by the HCP including FreeSurfer outputs.^1^ Total surface area A_t_ (pial surface area corrected for areas towards the corpus callosum), exposed surface area A_e_ (hull surface that envelops the pial surface but excluding sulcal regions corrected for areas towards the corpus callosum), and average cortical thickness T (distance between the white and the pial surface corrected for areas towards the corpus callosum) were extracted for each participant as described previously by Wang et al.^2^ To investigate the universal scaling law, we transformed to log coordinates, i.e., , x=log_10_(A_e_) and y=log_10_(A_t_T^1/2^) with α as a slope.

The slope of all healthy young adults was 1.22 (95%-CI=1.14-1.29). For male subjects, the slope was 1.21 (95%-CI=1.08-1.34). The theoretically predicted slope of 1.25 is within the 95%-CI of the slope for these subjects. For female subjects, the slope was 1.25 (95%-CI=1.11-1.40).

**
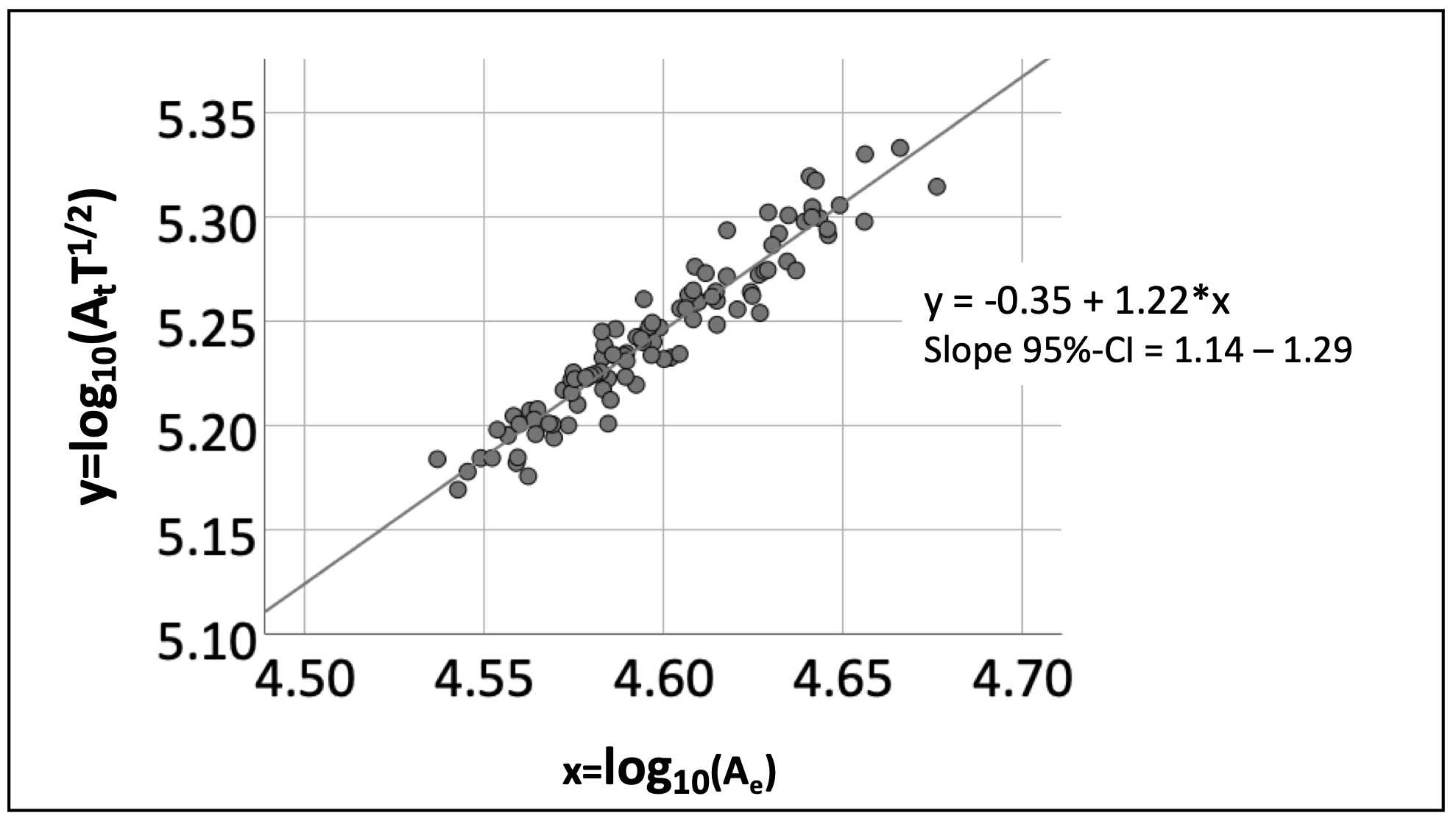
**

**Supplementary Figure 2: Scaling law in healthy young adults from the HCP cohort.** The relationship between x=log_10_(A_e_) and y=log_10_(A_t_T^1/2^) is shown as a scatter plot. The linear regression line and equation as well as the 95%-CI interval for the slope were added.

Abbreviations: A_e_, exposed surface area; A_t_, total surface area; T, cortical thickness.

**Supplementary Table 1: Comparison between VP/VLBW subjects with MRI data and without MRI data**

|  | **VP/VLBW with MRI (n=101)** | | **VP/VLBW without MRI (n=159)** | |  |
| --- | --- | --- | --- | --- | --- |
|  | **Mean** | **SD** | **Mean** | **SD** | **p value** |
| **GA (weeks)** | 30.5 | ± 2.1 | 30.6 | ± 2.3 | 0.656 |
| **BW (g)** | 1324 | ± 313 | 1323 | ± 320 | 0.980 |
| **Full-scale IQ^a^ (a.u.)** | 94.1 | ± 12.7 | 79.5 | ± 22.9 | **<0.001** |

Statistical comparisons: GA, BW and FS-IQ with two sample t-tests. Bold letters indicate statistical significance defined as p<0.05.

Abbreviations: BW, birth weight; GA, gestational age; IQ, intelligence quotient; SD, standard deviation; MRI, magnetic resonance imaging; VP/VLBW, very preterm and/or very low birth weight.

^a^ Data are based on 97 VP/VLBW subjects with MRI data and 120 VP/VLBW subjects without MRI data

**References**

1. Glasser MF, Sotiropoulos SN, Wilson JA, et al. The minimal preprocessing pipelines for the Human Connectome Project. *Neuroimage*. 2013;80:105-124. doi:10.1016/j.neuroimage.2013.04.127

2. Wang Y, Necus J, Kaiser M, Mota B. Universality in human cortical folding in health and disease. *Proc Natl Acad Sci*. 2016;113(45):12820-12825. doi:10.1073/pnas.1610175113
